# The impact of ammonia on particle formation in the Asian Tropopause Aerosol Layer

**DOI:** 10.1038/s41612-024-00758-3

**Published:** 2024-09-12

**Authors:** Christos Xenofontos, Matthias Kohl, Samuel Ruhl, João Almeida, Hannah M. Beckmann, Lucía Caudillo-Plath, Sebastian Ehrhart, Kristina Höhler, Milin Kaniyodical Sebastian, Weimeng Kong, Felix Kunkler, Antti Onnela, Pedro Rato, Douglas M. Russell, Mario Simon, Leander Stark, Nsikanabasi Silas Umo, Gabriela R. Unfer, Boxing Yang, Wenjuan Yu, Marcel Zauner-Wieczorek, Imad Zgheib, Zhensen Zheng, Joachim Curtius, Neil M. Donahue, Imad El Haddad, Richard C. Flagan, Hamish Gordon, Hartwig Harder, Xu-Cheng He, Jasper Kirkby, Markku Kulmala, Ottmar Möhler, Mira L. Pöhlker, Siegfried Schobesberger, Rainer Volkamer, Mingyi Wang, Stephan Borrmann, Andrea Pozzer, Jos Lelieveld, Theodoros Christoudias

**Affiliations:** 1 Climate and Atmosphere Research Center (CARE-C), The Cyprus Institute, Nicosia, Cyprus; 2 Department of Atmospheric Chemistry, Max Planck Institute for Chemistry, Mainz, Germany; 3 CERN, the European Organization for Nuclear Research, Geneva, Switzerland; 4 Faculty of Sciences of the University of Lisbon, Lisbon, Portugal; 5 Department of Environmental Physics, University of Tartu, Tartu, Estonia; 6 Institute for Atmospheric and Environmental Sciences, Goethe University Frankfurt, Frankfurt am Main, Germany; 7 Institute of Meteorology and Climate Research, Atmospheric Aerosol Research, Karlsruhe Institute of Technology, Karlsruhe, Germany; 8 Division of Chemistry and Chemical Engineering, California Institute of Technology, Pasadena, CA USA; 9 Institute of Ion Physics and Applied Physics, University of Innsbruck, Innsbruck, Austria; 10 Atmospheric Microphysics Department, Leibniz Institute for Tropospheric Research (TROPOS), Leipzig, Germany; 11 Laboratory of Atmospheric Chemistry, Paul Scherrer Institute, Villigen, Switzerland; 12 Institute for Atmospheric and Earth System Research/Physics, Faculty of Science, University of Helsinki, Helsinki, Finland; 13 TOFWERK, Thun, Switzerland; 14 IONICON Analytik GmbH, Innsbruck, Austria; 15 Department of Chemical Engineering, Carnegie Mellon University, Pittsburgh, PA USA; 16 Department of Engineering and Public Policy, Carnegie Mellon University, Pittsburgh, PA USA; 17 Department of Chemistry, Carnegie Mellon University, Pittsburgh, PA USA; 18 Center for Atmospheric Particle Studies, Carnegie Mellon University, Pittsburgh, PA USA; 19 Yusuf Hamied Department of Chemistry, University of Cambridge, Cambridge, United Kingdom; 20 Helsinki Institute of Physics, University of Helsinki, Helsinki, Finland; 21 Joint International Research Laboratory of Atmospheric and Earth System Sciences, School of Atmospheric Science, Nanjing University, Nanjing, China; 22 Aerosol and Haze Laboratory, Beijing Advanced Innovation Center for Soft Matter Science and Engineering, Beijing University of Chemical Technology, Beijing, China; 23 Faculty of Physics and Earth Sciences, Leipzig Institute for Meteorology, Leipzig University, Leipzig, Germany; 24 Department of Technical Physics, University of Eastern Finland, Kuopio, Finland; 25 Cooperative Institute for Research in Environmental Sciences, University of Colorado Boulder, Boulder, CO USA; 26 Department of the Geophysical Sciences, The University of Chicago, Chicago, IL USA; 27 Institute for Atmospheric Physics, Johannes Gutenberg University, Mainz, Germany; 28 Particle Chemistry Department, Max Planck Institute for Chemistry, Mainz, Germany

**Keywords:** Atmospheric chemistry, Atmospheric chemistry

## Abstract

During summer, ammonia emissions in Southeast Asia influence air pollution and cloud formation. Convective transport by the South Asian monsoon carries these pollutant air masses into the upper troposphere and lower stratosphere (UTLS), where they accumulate under anticyclonic flow conditions. This air mass accumulation is thought to contribute to particle formation and the development of the Asian Tropopause Aerosol Layer (ATAL). Despite the known influence of ammonia and particulate ammonium on air pollution, a comprehensive understanding of the ATAL is lacking. In this modelling study, the influence of ammonia on particle formation is assessed with emphasis on the ATAL. We use the EMAC chemistry-climate model, incorporating new particle formation parameterisations derived from experiments at the CERN CLOUD chamber. Our diurnal cycle analysis confirms that new particle formation mainly occurs during daylight, with a 10-fold enhancement in rate. This increase is prominent in the South Asian monsoon UTLS, where deep convection introduces high ammonia levels from the boundary layer, compared to a baseline scenario without ammonia. Our model simulations reveal that this ammonia-driven particle formation and growth contributes to an increase of up to 80% in cloud condensation nuclei (CCN) concentrations at cloud-forming heights in the South Asian monsoon region. We find that ammonia profoundly influences the aerosol mass and composition in the ATAL through particle growth, as indicated by an order of magnitude increase in nitrate levels linked to ammonia emissions. However, the effect of ammonia-driven new particle formation on aerosol mass in the ATAL is relatively small. Ammonia emissions enhance the regional aerosol optical depth (AOD) for shortwave solar radiation by up to 70%. We conclude that ammonia has a pronounced effect on the ATAL development, composition, the regional AOD, and CCN concentrations.

## Introduction

New particle formation (NPF) in the free troposphere is a predominant global source of cloud condensation nuclei (CCN)^1^, which are critical components in cloud formation and influence the climate^2,3^. This process begins with particle nucleation, which involves the spontaneous condensation of low-volatility vapours in the atmosphere, leading to liquid or solid particle formation^4^. Initial stable molecular clusters form with diameters just above 1 nm^5^. For these new particles to become CCN, they should not be scavenged by pre-existing aerosols and need to grow through further vapour condensation to a size of around 50 nm and larger^6^. However, NPF remains insufficiently understood, particularly in the cold upper troposphere and lower stratosphere (UTLS) over tropical convective regions^7,8^. This is due to the limited knowledge about the precursor vapours that contribute to forming particles. Current atmospheric models underrepresent these crucial NPF mechanisms, including the synergistic interaction of ammonia with nitric acid and sulphuric acid in the UTLS. This knowledge gap is apparent in regions affected by the Asian monsoon, which influences the climate and air quality for nearly half the global population^9^. Initiated by the surface cyclone, convective transport carries gaseous precursors from the boundary layer to the UTLS^10,11^. This convective activity, coupled with the circulation of the South Asian (summer) monsoon anticyclone, is thought to contribute to NPF and the development of an enhanced aerosol layer, called the Asian Tropopause Aerosol Layer (ATAL)^12–14^.

The ATAL, discovered through satellite and balloon measurements, extends from the Middle East to Eastern Asia and covers a vertical range from 11 to 19 km^15–17^. It forms in June with the onset of the monsoon and dissipates in September with the breakup of the anticyclonic circulation^17–21^. The composition of the ATAL has been a subject of scientific discourse for the past decade. Previous modelling studies have indicated that aerosols in the ATAL consist of sulphate, organics, nitrate, and ammonium^22–25^. Appel et al., using aircraft-borne in situ measurements, detected increased mass concentrations of particulate nitrate, ammonium, and organic compounds at altitudes between ~13 and 18 km in the South Asian monsoon region^17^. Höpfner et al. through satellite measurements and aircraft observations, report that convectively lifted ammonia contributes to the ATAL composition by forming ammonium aerosol particles^26,27^. However, the precise influence of ammonia in modulating the development, persistence, and composition of the ATAL aerosol species remains unresolved.

Ammonia constitutes nearly 50% of the total reactive nitrogen emissions into the atmosphere^28,29^. Almost 90% of global ammonia emissions originate from agriculture, including fertiliser use and livestock manure^30^. Other atmospheric ammonia sources include combustion-related emissions^31^, industrial processes^32^, and volatilisation from soils and oceans^33^. Asian emissions account for about 50% of global ammonia emissions and contribute notably to air pollution^34,35^. Recent satellite observations have revealed enhanced amounts of ammonia, with concentrations reaching up to 30 pptv, in the South Asian summer monsoon UTLS^26^. Previous modelling studies indicate that accurate estimations of ammonia emissions are crucial for predicting future concentrations of ammonium and nitrate aerosols in the UTLS^36^. Ammonium nitrate aerosols provide additional particle surfaces that scatter incoming shortwave solar radiation and, therefore, affect the radiative balance of Earth^37^. Future projections indicate that ammonia emissions in India could double by 2050, which highlights an urgent need for research into its influence on particle formation^38–40^.

This paper examines the link between ammonia and particle formation within the South Asian monsoon UTLS. We use parameterisations of NPF derived from experiments conducted at the CERN CLOUD (Cosmics Leaving OUtdoor Droplets) chamber^41–47^ to explore the synergistic effects of ammonia with nitric acid and sulphuric acid under upper tropospheric conditions. By incorporating these parameterisations into the state-of-the-art EMAC (ECHAM/MESSy Atmospheric Chemistry) climate-chemistry model^48,49^, we analyse the contributions of individual nucleation pathways to the overall nucleation rate and assess the impact of ammonia on the ATAL through simulations comparing current and zero ammonia emissions scenarios. For the latter scenario, the ammonia emissions are switched off globally to isolate their influence on the ATAL, eliminating transboundary pollution effects. All other conditions remain constant to ensure any observed differences result solely from the change in ammonia emissions. Existing emissions inventories lack the accuracy of ammonia source data for the Indian subcontinent to be effectively used in atmospheric models^50^. In particular, the nitrogen excretion rates and ammonia emissions rates for manure from animal houses and storage systems are the main input parameters causing this uncertainty^51^. Our sensitivity analysis aims to encompass the broad uncertainty range in ammonia emissions. We aim to determine the efficiency of convective ammonia transport and its influence on NPF, as well as the mass and chemical composition of the ATAL. Finally, we will quantify the effect of ammonia-driven particle formation and growth on the regional aerosol optical depth and CCN concentrations.

## Results

### Model evaluation

We compare the simulated aerosol vertical profiles generated by the EMAC model against the StratoClim (Stratospheric and upper tropospheric processes for better Climate predictions) airborne field campaign observations over the South Asian monsoon region between 27 July and 10 August 2017. During StratoClim, aircraft in situ measurements were performed of the chemical composition of the ATAL and particle number concentrations across eight flights^17,27^.

**Fig. 1:**
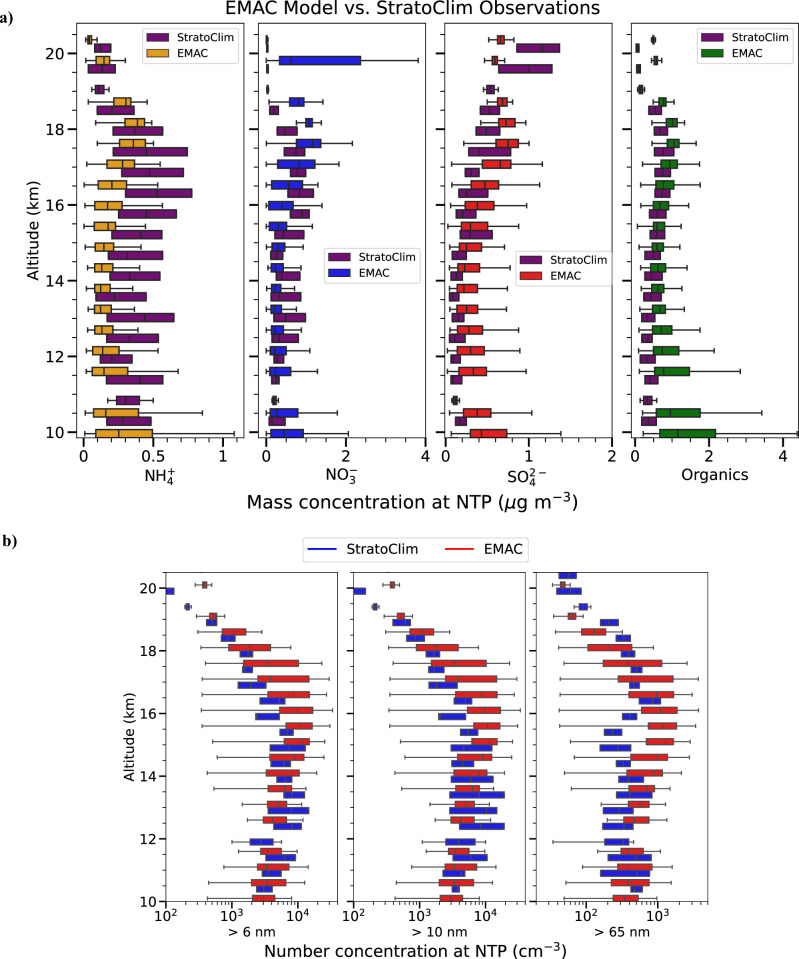
Comparison of EMAC model aerosol mass and number concentrations with StratoClim 2017 observations. **a** Vertical profiles of aerosol constituents in the South Asian monsoon region (10–50^∘^N, 60–110^∘^E) for particle sizes between 0.09 and 1 μm. Model data are compared to all eight flights during StratoClim between 27 July and 10 August 2017. Boxplots represent the distribution of observed (StratoClim) and modelled (EMAC) mass concentrations of ${\,\text{NH}\,}_{4}^{+}$, ${\,\text{NO}\,}_{3}^{-}$, ${\,\text{SO}\,}_{4}^{2-}$, and organics in the ATAL. The central line of each boxplot denotes the median value, while the box boundaries indicate the interquartile range. The whiskers represent the range limits. **b** Vertical profiles of aerosol particle number concentrations in EMAC (red) and StratoClim (blue) over the South Asian monsoon region (10–50^∘^N, 60–110^∘^E) as a function of altitude for particle size greater than 6 nm, 10 nm, and 65 nm. The boxplots follow the same conventions as in (**a**). Both particle mass and number concentrations are referenced at normal temperature and pressure (NTP).

In the course of the simulation, model data are sampled at the model grid boxes along the actual flight tracks, including specific flight dates and times, to ensure close correspondence with observed measurements. Figure 1a shows a direct comparison of the simulated vertical distribution of aerosol mass concentrations in the ATAL with the observations from the StratoClim campaign for particle sizes between 0.09 and 1 μm. This size range aligns with the detection capabilities of the aerosol mass spectrometer used during the StratoClim campaign^17^. The comparison of model data and observations includes a composite of all eight StratoClim flights. The boxplots show the variations in the PM_1_ (particulate matter less than 1 μm) mass concentrations of ammonium (${\,\text{NH}\,}_{4}^{+}$), nitrate (${\,\text{NO}\,}_{3}^{-}$), sulphate (${\,\text{SO}\,}_{4}^{2-}$), and organic particles in the ATAL. There is a good agreement between the observations and the model outputs. The vertical profiles show clear enhancements in the mass concentration of aerosols, particularly for organic particles, ${\,\text{NO}\,}_{3}^{-}$, and ${\,\text{NH}\,}_{4}^{+}$ at altitudes between 15 and 18 km. Figure 1b illustrates a further comparison of the number concentrations for particle sizes greater than 6 nm, 10 nm, and 65 nm in the ATAL between EMAC and StratoClim. The EMAC model outputs are in good agreement with the StratoClim measurements.

Further evaluations with additional observations from StratoClim are provided in Supplementary Fig. 1. In particular, the relative humidity, atmospheric temperature, wind speed, water vapour, ozone (O_3_), and carbon monoxide (CO) mixing ratios in EMAC are evaluated and found to be in good agreement with StratoClim. Supplementary Fig. 2 illustrates the monthly progression of the South Asian (summer) monsoon anticyclone and the associated distribution of total particle number concentration in maps as simulated by the EMAC model, contributing to the assessment of the modelled dynamics. In previous studies, the EMAC model has also been widely applied and assessed compared to measurements of trace gases and aerosols from ground stations, aircraft, and satellites in both the troposphere and stratosphere^52–57^. Gottschaldt et al. report that their EMAC simulations accurately capture the reduction in the O_3_ mixing ratios within the South Asian monsoon anticyclone for July and August at 100 hPa by comparing them to aircraft observations^58,59^. Finally, Ojha et al. suggest that the EMAC model is capable of reproducing enhanced O_3_ concentrations in the upper troposphere over the Himalayas by comparing to ozone-sonde measurements^60^.

### Convection and new particle formation

Previous findings indicate that convection influences the distribution of aerosols and their precursors across different atmospheric layers in the South Asian monsoon region^13,61^. Our simulations compare the median nucleation rates in the ATAL, within the UTLS, between composites of days with convection and those without, for the two ammonia (NH_3_) emissions scenarios studied.

**Fig. 2:**
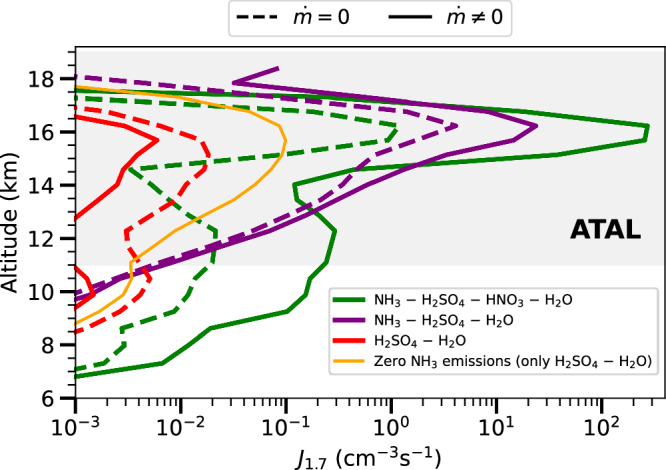
Simulated nucleation rates and the role of convection in the ATAL. Simulated nucleation rates at 1.7 nm (*J*_1.7_), calculated at ambient temperature and pressure, are shown at varying altitudes over the South Asian monsoon region (10–50^∘^N, 60–110^∘^E) for summer 2017. The nucleation mechanisms included are: synergistic NH_3_–H_2_SO_4_–HNO_3_–H_2_O (green); ternary NH_3_–H_2_SO_4_–H_2_O (purple); binary H_2_SO_4_–H_2_O under zero NH_3_ emissions (orange); and binary H_2_SO_4_–H_2_O in the presence of NH_3_ emissions (red). The solid lines (updraft mass flux rate $(\dot{m})\ne 0$) denote conditions with mass updraft, indicative of active deep convection, whereas the dashed lines $(\dot{m}=0)$ represent instances of a quiescent atmosphere without convection. The grey-shaded area denotes the ATAL region and coincides with the altitude range where the *J*_1.7_ is significantly influenced by the presence of convective updrafts.

Figure 2 illustrates the EMAC model simulation results for nucleation rates at 1.7 nm (*J*_1.7_) over the South Asian monsoon in the summer of 2017 for composites of days with deep convection (updraft mass flux rate $(\dot{m})\ne 0$) compared to those with no convection ($\dot{m}=0$). We find a strong positive effect of convection on NPF, particularly within the ATAL. Our simulation results suggest that the presence of NH_3_, sulphuric acid (H_2_SO_4_), nitric acid (HNO_3_), and water vapour (H_2_O) leads to the highest *J*_1.7_ at altitudes between 15 and 17 km under convective conditions (solid green line). The synergistic effect of all these species markedly enhances NPF^44,62^, especially concomitant with vertical transport mechanisms through convection^63^. Without convection in our simulations, the peak *J*_1.7_ of synergistic NH_3_–H_2_SO_4_–HNO_3_–H_2_O nucleation drops by two orders of magnitude (dashed green line), while for ternary NH_3_–H_2_SO_4_–H_2_O nucleation, the decrease is one order of magnitude (dashed purple line). The peak *J*_1.7_ of H_2_SO_4_–H_2_O nucleation is about three times larger than that simulated in the presence of convection. This is likely due to the reduced amount of convectively lifted NH_3_, which is predominantly consumed in ternary and synergistic nucleation mechanisms. As a consequence, there is a reduction in the participation of H_2_SO_4_ in these NH_3_-driven nucleation mechanisms, while its involvement in H_2_SO_4_–H_2_O nucleation is markedly enhanced.

In the scenario with zero NH_3_ emissions (orange line in Fig. 2), solely H_2_SO_4_ and H_2_O participate in nucleation. The peak *J*_1.7_ is three orders of magnitude lower than that with synergistic nucleation, and two orders of magnitude lower than ternary nucleation. We highlight the critical role of NH_3_ in the aerosol formation process under deep convective events.

### Diurnal cycle

Besides mass uplift by convection, the presence of sunlight has a strong influence on the diurnal variability of the *J*_1.7_ and particle growth within the ATAL. Recent studies have highlighted the substantial influence of diurnal heating and nocturnal cooling on the monsoon circulation and precipitation patterns^64,65^. Through a combination of model calculations and in situ measurements, Weigel et al.^66^ found that NPF exhibits diurnal variation in West Africa and Brazil in the absence of NH_3_ emissions but in the presence of mesoscale convective systems, which are also prevalent in the South Asian monsoon region.

**Fig. 3:**
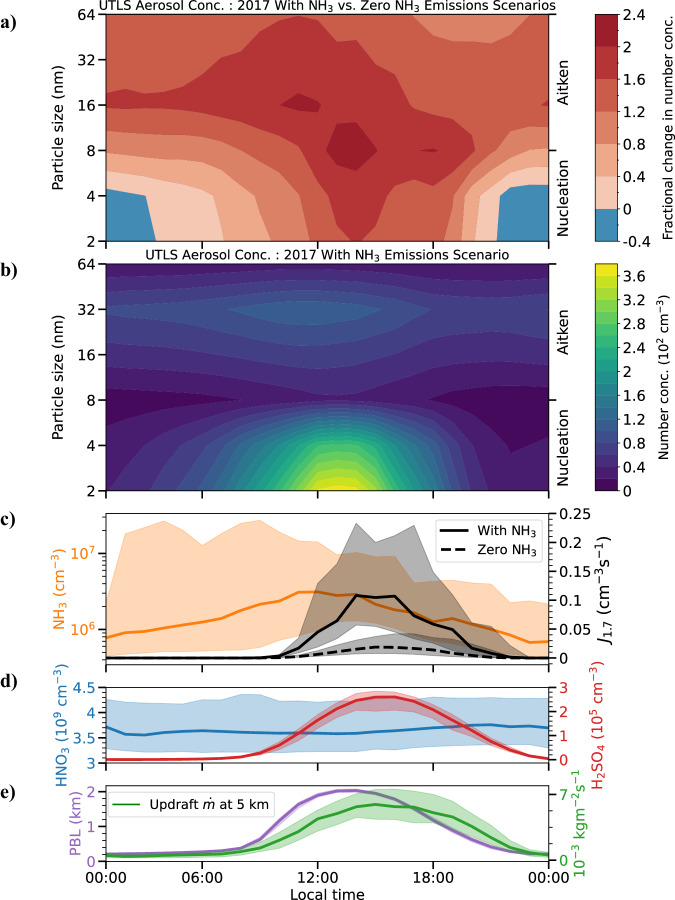
Simulated diurnal profile. The diurnal cycle for summer 2017 in the South Asian monsoon upper troposphere and lower stratosphere (UTLS) is shown. **a** Fractional change in particle number concentration resulting from the presence of NH_3_ relative to zero NH_3_ emissions, across particle sizes ranging from 2 to 64 nm. The red contours indicate greater fractional changes in concentration, signifying the role of NH_3_ in particle formation within the nucleation and Aitken modes. **b** Absolute particle number concentration for the 2017 scenario with NH_3_ emissions. **c** Diurnal variation in NH_3_ concentration and nucleation rate at 1.7 nm (*J*_1.7_) with (solid line) and without (dashed line) NH_3_ emissions in the UTLS, showing a 10-fold increase in the peak *J*_1.7_ with NH_3_ emissions. **d** H_2_SO_4_ and HNO_3_ concentrations in the UTLS in the presence of NH_3_ emissions. **e** Planetary boundary layer (PBL) height and updraft mass flux rate ($\dot{m}$) due to deep convection at 5 km altitude, where vertical velocities are close to maximum. All concentrations and *J*_1.7_ are calculated at ambient temperature and pressure. The lines and the shaded areas represent the medians and the interquartile range for the corresponding hour, respectively.

Our findings indicate a significant diurnal variation in the formation and growth of particles within the ATAL. The EMAC model output indicates the variation across different particle sizes, from nucleation (2–8 nm) to Aitken (16–64 nm) modes (Fig. 3a). Particle formation and growth are greatly enhanced during daylight hours when comparing aerosol number concentration between the scenario including NH_3_ emissions, with zero NH_3_ emissions.

Furthermore, as shown in Fig. 3b, the absolute aerosol concentration with NH_3_ emissions reveals that during daylight, smaller particles dominate due to nucleation events. A shift towards larger particle sizes is observed as the day progresses, with a continuous increase in the Aitken-size particle number concentration resulting from growth and the corresponding decrease in nucleation-mode particle numbers until a nocturnal decline occurs.

Thus, our simulations reveal a strong diurnal variation in the NPF rate within the ATAL, driven by NH_3_. During daytime, the peak in the *J*_1.7_ shows a 10-fold increase in scenarios with NH_3_ emissions compared to those without (Fig. 3c). This increase coincides with peaks in NH_3_ concentration, convection rates ($\dot{m}$), and H_2_SO_4_ concentration (Fig. 3d, e), suggesting that the peak in the *J*_1.7_ is linked to the availability of NH_3_ and H_2_SO_4_, key precursors for these particle formation processes, which are enhanced by convection that facilitates the vertical transport and mixing of precursor gases.

Acknowledging the critical roles of precursor gas availability and convection in governing diurnal variations in particle nucleation within the ATAL, it is important to understand how NH_3_ contributes not just to nucleation (determining the number concentration) but also to the mass concentration of particles and hence the chemical composition of the ATAL.

### Influence of NH_3_ on the ATAL composition

**Fig. 4:**
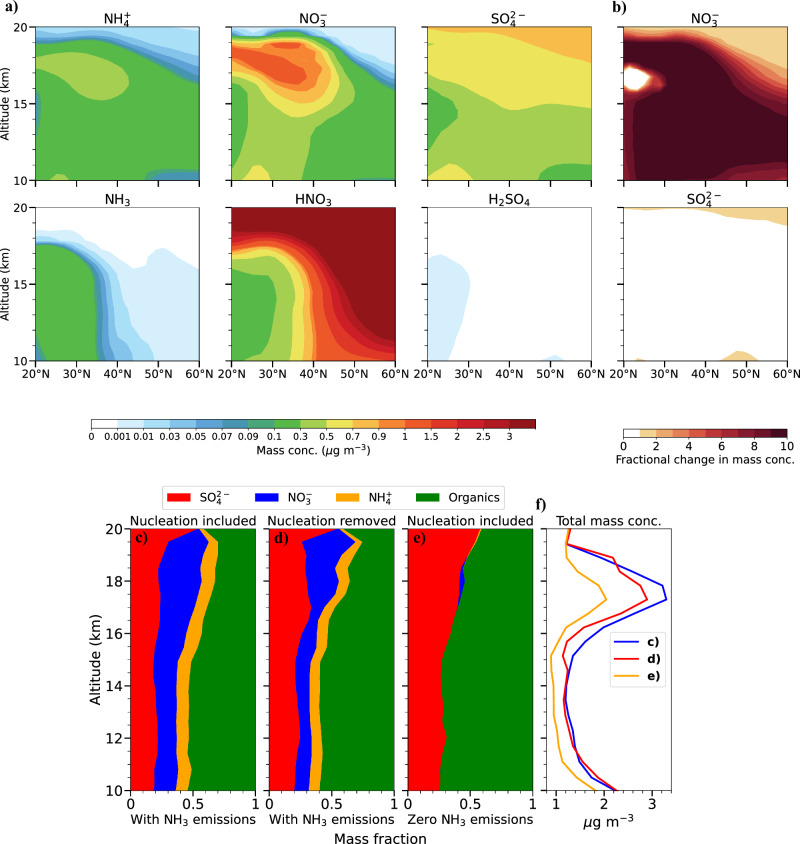
NH_3_ influence on the ATAL chemical composition. **a** Simulated zonal-averaged aerosol (top) and precursor gases (bottom) mass concentrations averaged over the summer of 2017 in the presence of NH_3_ emissions. **b** Simulated zonal-averaged profiles on the fractional change in the mass concentration of ${\,\text{NO}\,}_{3}^{-}$ (top) and ${\,\text{SO}\,}_{4}^{2-}$ (bottom) in June-July-August averaged for the year 2017 due to normal NH_3_ emissions levels relative to zero NH_3_ emissions. Simulated vertical profiles of the cumulative mass fraction of particulate organics (green), ${\,\text{NO}\,}_{3}^{-}$ (blue), ${\,\text{SO}\,}_{4}^{2-}$ (red), and ${\,\text{NH}\,}_{4}^{+}$ (orange) as a function of altitude for (**c**) NH_3_ emissions with all nucleation mechanisms included, (**d**) NH_3_ emissions without synergistic NH_3_–H_2_SO_4_–HNO_3_–H_2_O and ternary NH_3_–H_2_SO_4_–H_2_O nucleation mechanisms, and (**e**) zero NH_3_ emissions with all nucleation mechanisms included. **f** Vertical profiles of the total aerosol mass concentration corresponding to the scenarios in (**c**–**e**). All concentrations are referenced at normal temperature and pressure (NTP).

We use the EMAC model to investigate the impact of NH_3_ on the ATAL composition by contrasting scenarios with and without NH_3_ emissions during the summer of 2017. Figure 4a shows the simulated zonal-averaged profiles for aerosols and their precursors across the South Asian monsoon region in the presence of NH_3_ emissions, indicating higher abundances of NH_3_ and HNO_3_ than that of H_2_SO_4_. Lightning produces nitrogen oxides (NO_*x*_ = NO + NO_2_), which are oxidised to form HNO_3_. However, the local HNO_3_-forming reaction is not dominant in the upper tropospheric monsoon anticyclone. Other processes, notably transport within the UTLS region, also contribute substantially to the levels of HNO_3_ in the upper troposphere^11^. H_2_SO_4_ is primarily produced by the oxidation of sulphur dioxide (SO_2_)^67^. We quantify the impact of NH_3_ on ${\,\text{NO}\,}_{3}^{-}$ and ${\,\text{SO}\,}_{4}^{2-}$ levels by calculating the fractional change in the mass concentration of ${\,\text{NO}\,}_{3}^{-}$ and ${\,\text{SO}\,}_{4}^{2-}$ attributable to NH_3_ emissions (Fig. 4b). Our results show that, in conditions where HNO_3_ is relatively high, especially in the UTLS, excess NH_3_ reacts with it to form ammonium nitrate (NH_4_NO_3_). This results in a 10-fold increase in ${\,\text{NO}\,}_{3}^{-}$ levels. Our model calculates the molality of semi-volatile species and the equilibrium states of binary solutions, accounting for stable and metastable phases^68^. NH_4_NO_3_, being semi-volatile, evaporates at the higher temperatures found in the lower troposphere but remains in the particulate phase in the UTLS^27^. This property can profoundly influence its contribution to particle growth and composition in the ATAL.

Exploring the influence of NH_3_ on the ATAL elucidates its significant role in modulating aerosol composition via NPF and subsequent growth. We performed a sensitivity test to isolate the influence of nucleation mechanisms involving NH_3_ on aerosol mass concentrations. This test was executed in the absence of nucleation events involving NH_3_, despite the presence of NH_3_ emissions. Figure 4c–e shows the simulated vertical profiles of the cumulative mass fraction of particulate organics, ${\,\text{NO}\,}_{3}^{-}$, ${\,\text{SO}\,}_{4}^{2-}$, and ${\,\text{NH}\,}_{4}^{+}$ as a function of altitude for the summer of 2017. Figure 4c includes all nucleation mechanisms applied in the model. This means that it considers all known interactions between NH_3_, H_2_SO_4_, HNO_3_, and H_2_O in forming new particles. This scenario includes NH_3_ emissions. Figure 4d also includes NH_3_ emissions but excludes the synergistic NH_3_–H_2_SO_4_–HNO_3_–H_2_O and ternary NH_3_–H_2_SO_4_–H_2_O nucleation mechanisms from the model run. Figure 4e illustrates the scenario with zero NH_3_ emissions, which includes all the nucleation mechanisms considered in the model. Organics constitute ~40% of the simulated ATAL mass. Secondary organic aerosols derived from volatile organic compounds constitute ~90% of the total organic mass in the ATAL. In contrast, primary organic aerosols, originating from biomass and fossil fuel combustion, account for the remaining 10%^69^. ${\,\text{NO}\,}_{3}^{-}$ contributes to around 30% of the simulated ATAL mass, while ${\,\text{SO}\,}_{4}^{2-}$ and ${\,\text{NH}\,}_{4}^{+}$ make up about 20% and 10% of the total mass, respectively. Removing synergistic and ternary nucleation mechanisms reduces the ${\,\text{NO}\,}_{3}^{-}$ mass fraction by ~10% below 16 km altitude relative to the case where all nucleation mechanisms are included. This decrease reaches a maximum of 20% around 16 km altitude. The mass fraction of ${\,\text{SO}\,}_{4}^{2-}$ increases by approximately the same amount due to the reaction of NH_3_ with H_2_SO_4_ to form ammonium sulphate (NH_4_)_2_SO_4_. The ${\,\text{NH}\,}_{4}^{+}$ mass fraction remains relatively stable. With zero NH_3_ emissions, ${\,\text{SO}\,}_{4}^{2-}$ and organics comprise almost all of the simulated ATAL mass. The ${\,\text{NH}\,}_{4}^{+}$ and ${\,\text{NO}\,}_{3}^{-}$ mass fractions are almost completely diminished relative to the case where NH_3_ emissions are included.

Figure 4f illustrates the vertical profiles of the total aerosol mass concentration in the ATAL as a function of altitude. The profiles correspond to the scenarios shown in Fig. 4c–e. When all nucleation mechanisms and NH_3_ emissions are included, the mass concentration is the highest among the three scenarios. A 10% reduction in the total mass concentration is observed at altitudes between 15 and 18 km when nucleation events lack the contribution of NH_3_, even though NH_3_ is present. Lastly, the lowest mass concentration is observed with zero NH_3_ emissions across the entire altitude range. This reduction in mass concentration reaches a maximum of 40% around 17 km altitude relative to the scenario with NH_3_ emissions.

Our results show that eliminating NH_3_ involvement in nucleation leads to a change in ${\,\text{NO}\,}_{3}^{-}$ and ${\,\text{SO}\,}_{4}^{2-}$ mass fractions in the ATAL (Fig. 4d). These changes are smaller than in scenarios without NH_3_ emissions (Fig. 4e). This result agrees with Höpfner et al.^27^, who suggest that NH_3_ enhances NH_4_NO_3_ formation in the ATAL. However, our findings suggest that there is a relatively small impact of NH_3_-driven NPF on mass concentration.

### Effects on the regional AOD and CCN

We model the concentrations of CCN at 0.2% supersaturation (CCN_0.2%_) and the aerosol optical depth (AOD) at 550 nm (shortwave) for the aforementioned cases with and without NH_3_ emissions.

**Fig. 5:**
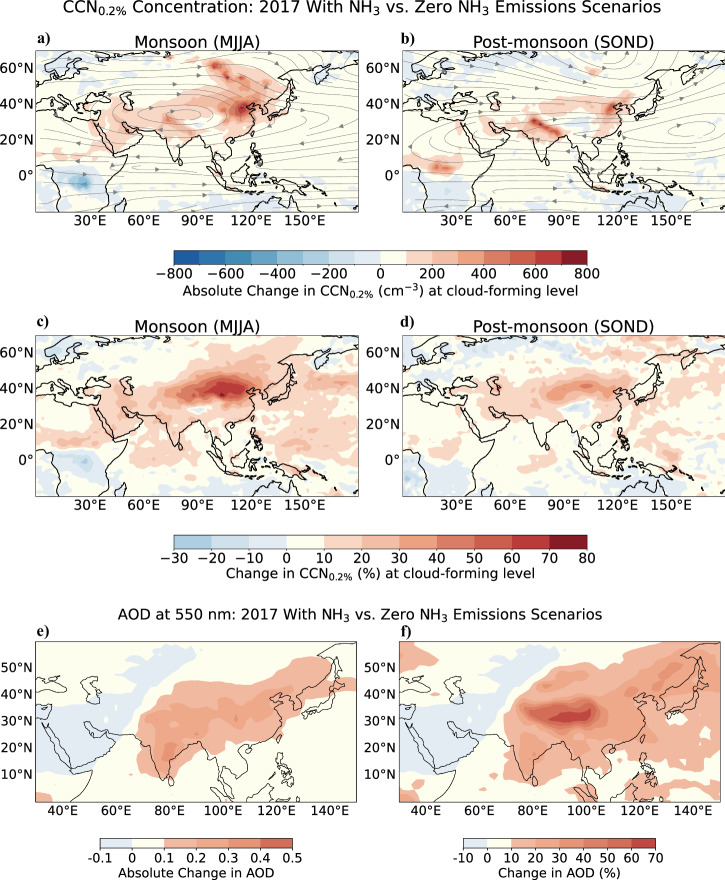
Influence of NH_3_ on the regional AOD and CCN in the EMAC model. Variations in CCN at 0.2% supersaturation (CCN_0.2%_) and aerosol optical depth (AOD) are shown for NH_3_ emissions vs. zero NH_3_ emissions scenarios. Absolute change in CCN_0.2%_ (cm^−3^) at cloud-forming level between the different NH_3_ emissions scenarios during the (**a**) South Asian monsoon (MJJA) and (**b**) post-monsoon (SOND) seasons. The grey arrows indicate the wind direction. Percentage change in CCN_0.2%_ between the scenarios with and without NH_3_ at cloud-forming level for the (**c**) monsoon (MJJA) and (**d**) post-monsoon (SOND) seasons. All concentrations are calculated at ambient temperature and pressure. Simulated spatial distribution of (**e**) absolute and (**f**) percentage changes in total column AOD at 550 nm when comparing the 2017 NH_3_ emissions to a zero NH_3_ emissions scenario during the monsoon season.

NH_3_ has a pronounced influence on NPF in the ATAL (Fig. 3). After continued growth, these newly formed particles follow descending air masses into the lower troposphere. At these lower altitudes, they can become an important CCN source^2^. We find that NH_3_ emissions lead to significant seasonal variations in CCN_0.2%_ concentrations across the South Asian monsoon region. Figure 5a–d illustrates the difference in CCN_0.2%_ concentrations at the model convective cloud base level, when comparing the 2017 NH_3_ emissions to a zero NH_3_ emissions scenario in EMAC. This level represents the altitude at which clouds form. CCN_0.2%_ outflow from Central Asia is substantial as air flows diverge and streamlines suggest eastward transport. In comparison to the scenario with zero NH_3_ emissions, we observe an increase in CCN_0.2%_ concentrations at cloud-forming level of up to 80%, corresponding to a maximum concentration of 800 cm^−3^ when NH_3_ emissions are included. This finding highlights the role of NH_3_ in cloud processes over the region.

The influence of NH_3_ on particle formation extends to the overall aerosol mass concentration and chemical composition within the ATAL, which affect the AOD and contribute to its modifications^25^. Figure 5e, f shows the simulated spatial distribution of the total atmospheric column changes in AOD at 550 nm for the different NH_3_ emissions scenarios over the monsoon summer period. NH_3_ emissions increase the aerosol mass concentration over the South Asian monsoon region (Fig. 4) and, therefore, increase the AOD at 550 nm by as much as 0.5, equivalent to 70%.

## Discussion

This study investigates the effects of NH_3_ on particle formation in the UTLS of the South Asian monsoon region. We use the ECHAM/MESSy Atmospheric Chemistry (EMAC) model to compare scenarios with and without NH_3_ emissions for the year 2017. NH_3_ is identified as a significant contributor to particle formation and growth in the South Asian monsoon region, and affects the composition of the ATAL.

Our model simulations show that NH_3_ enhances NPF rates by 10-fold during daytime due to vertical transport via deep convection over the region, compared to a baseline scenario of zero NH_3_ emissions (Fig. 3). This process significantly influences the particle size distribution and number concentration in the UTLS. Our analysis reveals that the influence of NH_3_ on aerosol mass concentrations and chemical composition is substantial through particle growth in the ATAL, as is evidenced by an order of magnitude increase in ${\,\text{NO}\,}_{3}^{-}$ levels with NH_3_ emissions (Fig. 4). NPF driven by NH_3_ has a relatively minor effect on aerosol mass and composition in the ATAL. Specifically, we find that removing the mechanisms for nucleation involving NH_3_ reduces the total aerosol mass concentration by 10% at altitudes between 15 and 18 km. This reduction reaches a maximum of 40% around 17 km altitude when NH_3_ emissions are removed.

Our results indicate substantial influence of particle formation in the ATAL on the regional AOD and CCN concentrations. There is a marked increase in CCN concentrations to a maximum of 800 cm^−3^, equivalent to 80% (Fig. 5), which is attributed predominantly to particle formation and growth driven by NH_3_. This increase in CCN, which is seen at cloud-forming heights, directly affects cloud formation. Furthermore, we observe an increase in AOD to a maximum of 0.5, equivalent to a 70% increase with NH_3_ relative to zero NH_3_ emissions.

Our study opens future research directions, such as expanding the geographical analysis to understand the impact of NH_3_ globally and incorporating these findings to refine the predictive accuracy of global climate projection models. Although our primary analysis is on the regional AOD and CCN, we recognise the importance of linking these changes to broader climate effects such as radiative forcing and subsequent temperature and precipitation changes. Future work will expand on these findings to quantify the radiative forcing associated with NH_3_-induced changes in the ATAL and the resultant regional climate impacts. It is critical to align our findings with anticipated NH_3_ emissions outlined in the IPCC (Intergovernmental Panel on Climate Change) scenarios^70,71^, thus providing a more comprehensive understanding of the role of NH_3_ in future climate.

## Methods

### EMAC model configuration

The EMAC model is a numerical simulation framework for global chemistry and climate interactions that includes submodels that describe processes in the atmosphere and their exchanges with oceans, lands, and anthropogenic factors^72^. The core atmospheric circulation model ECHAM5 is coupled with the second version of the Modular Earth Submodel System (MESSy2) to link multi-institution computer codes^48^. The meteorological prognostic variables are nudged through Newtonian relaxation towards the ECMWF ERA-5 reanalyses to ensure realistic simulation of transport conditions for selected periods for which model results are to be compared with atmospheric measurements. For each model time step, atmospheric chemical kinetics are calculated online using the MIM chemistry mechanism^73^, evaluated^74^ and described previously for use in global climate-chemistry simulations^53,54^.

We use EMAC (ECHAM5 version 5.3.02, MESSy version 2.55.2) in the T63L90 resolution and cover the period from January 2017 to January 2020, preceded by a decade-long spin-up simulation. Supplementary Fig. 3 compares the variability of model outputs across different years in EMAC. We observe minimal interannual variability, which underscores 2017 as an indicative year. We specify 90 vertical hybrid levels from the surface up to ~80 km altitude (0.01 hPa) and a spherical truncation of T63, which equates to a grid resolution of 1.875^∘^ by 1.875^∘^ for both latitude and longitude at the equator. Trace gas emissions, and NH_3_ in particular, are taken from the Community Emissions Data System^75^. The spatial distribution and intensity of these simulated NH_3_ emissions during the South Asian monsoon are illustrated in Supplementary Fig. 4. A time step of 10 min is used, and the output is saved every hour. In our simulations, the submodels used include: (i) GMXe for aerosol microphysics^76^, (ii) NAN for the nucleation mechanisms^77^, (iii) IONS for ion pair production rates from galactic cosmic rays and radon decay^77^, (iv) AEROPT for aerosol optical properties^78^, (v) MECCA for gas phase chemistry^79^, (vi) JVAL for photochemistry^80^, (vii) SCAV for the absorption of SO_2_, HNO_3_, and NH_3_, and the wet deposition of gases and aerosols^81^, (viii) DRY-DEP for dry deposition^82^, and (ix) SEDI for aerosol sedimentation^82^.

In this study, the CONVECT submodel is used for parameterising convection. The Tiedtke scheme^83^ with Nordeng closure^84^ is used as a standard setting employed for T63 resolution^56^. NO_*x*_ emissions from lightning activity are computed in real-time using the LNOX submodel^85^. The parameterisation developed by Grewe et al. ^86^, which correlates flash frequency with updraft velocity, is applied in this study. The convection^56,83,84^ and lightning^86^ parameterisation schemes used in this study have been previously evaluated^58,85,87^, showing particularly good agreement for the South Asian monsoon region^58^.

Regarding our diurnal cycle analysis, we have mitigated against any potential short-term build-up of pollutants in our simulations, which could dampen the diurnal cycle, by implementing a long spin-up period of 10 years. Further, precursor gases such as NH_3_ are depleted during the diurnal cycle and can only be replenished by transport. Any accumulation of pollutants does not survive the diurnal cycle due to convection and/or transport. Our model includes full photochemistry with reaction rates calculated online using JVAL^80^.

### NAN and IONS submodels

NPF in the EMAC nucleation mode is treated by the NAN (New Aerosol Nucleation) submodel^77^. NAN calculates nucleation rates based on the nucleation parameterisations published by the CERN CLOUD experiment: (i) binary H_2_SO_4_–H_2_O^41^, (ii) ternary NH_3_–H_2_SO_4_–H_2_O^41^, and (iii) synergistic NH_3_–H_2_SO_4_–HNO_3_–H_2_O^44^. A brief overview of the parameterisations is provided here, while the specifics, including the selection of functions, the number of parameters, and optimisation, are elaborated in the supplementary information of the aforementioned studies. The implementation of the NPF parameterisations used in EMAC is explained in ref. ^77^.

The neutral binary homogeneous nucleation involving H_2_SO_4_ and H_2_O is given by

$${J}_{b,n}={k}_{b,n}(T){\left[{\text{H}}_{2}{\text{SO}}_{4}\right]}^{{p}_{b,n}},$$

where *p* is a fitting parameter. The neutral homogeneous ternary nucleation of NH_3_–H_2_SO_4_–H_2_O is given by

$${J}_{t,n}={k}_{t,n}(T){f}_{n}\left(\left[{\text{H}}_{2}{\text{SO}}_{4}\right],\left[{\text{NH}}_{3}\right]\right).$$

The indices denote the type of nucleation: *b* for binary, *t* for ternary, *n* for neutral, and *i* for ion-induced nucleation. The function *k*_*x*,*y*_(*T*) shows the dependency of NPF on temperature, *T*, in Kelvin. It maintains a consistent form for the binary and ternary nucleation pathways and is expressed as

$$\ln {k}_{x,y}(T)={u}_{x,y}-\exp \left({v}_{x,y}\left(\frac{T}{1,000\,\,\text{K}\,}-{w}_{x,y}\right)\right),$$

where *u*, *v*, and *w* are fitting coefficients, with *x* ∈ (*b*, *t*), and *y* ∈ (*n*, *i*). The saturation behaviour of the ternary nucleation is controlled by

$${f}_{y}([{{\rm{H}}}_{2}{{\rm{SO}}}_{4}],[{{\rm{NH}}}_{{\rm{3}}}])=\frac{{[{{\rm{H}}}_{2}{{\rm{SO}}}_{4}]}^{{p}_{t,y}}[{{\rm{NH}}}_{{\rm{3}}}]}{{a}_{y}+\frac{{[{{\rm{H}}}_{2}{{\rm{SO}}}_{4}]}^{{p}_{t,y}}}{{[{{\rm{NH}}}_{{\rm{3}}}]}^{{p}_{A,y}}}},$$

where *a* and *p* are fitting parameters. This function is shared with the ion-induced ternary nucleation pathway. The equations for neutral nucleation are multiplied by the concentration of negative ions, [*n*^−^], to derive the equations for ion-induced nucleation. This results in

$${J}_{b,i}={k}_{b,i}(T)\left[{n}^{-}\right]{\left[{\text{H}}_{2}{\text{SO}}_{4}\right]}^{{p}_{b,i}},$$

and

$${J}_{t,i}={k}_{t,i}(T)\left[{n}^{-}\right]{f}_{i}\left(\left[{\text{H}}_{2}{\text{SO}}_{4}\right],\left[{\text{NH}}_{3}\right]\right).$$

Dunne et al. ^41^ derived a scaling factor for relative humidity that varies with *T*. However, this is based on very few measurements and its effect is relatively small. Therefore, the relative humidity scaling factor is not used here.

The parameterisation for the synergistic nucleation^44^ is given by

$${J}_{1.7}=2.9\times 1{0}^{-98}\exp \left(\frac{14,000}{T}\right){[{{\rm{H}}}_{2}{{\rm{SO}}}_{4}]}^{3}{[{{\rm{HNO}}}_{{\rm{3}}}]}^{2}{[{{\rm{NH}}}_{{\rm{3}}}]}^{4},$$

where the concentration of the precursor gases (H_2_SO_4_, HNO_3_, NH_3_), is given in molecules per cm^3^. Given that the experiments for synergistic nucleation were conducted exclusively at 223 K and previous studies have indicated that synergistic nucleation is undetectable at higher temperatures^63^, we assume that the parameterisation and the temperature-dependence function should be applied only to temperatures below 248 K. For higher temperatures, *J*_1.7_ is set to zero, with a smooth transition implemented near 248 K to avoid sudden changes.

The IONS submodel calculates atmospheric ion pair production rates and steady-state concentrations, accounting for galactic cosmic rays and radon decay. It provides online calculations of ion pair production rates for ion-induced nucleation while accounting for ion pair losses through ion-ion recombination and uptake by aerosol particles. Both NAN and IONS submodels have been evaluated in ref. ^77^.

### Aerosol representation in EMAC

The GMXe (Global Modal-aerosol eXtension) submodel^76^ integrates aerosol dynamics through a full thermodynamic treatment of gas/aerosol partitioning with the ISORROPIA-II model^68^, and treats the aerosol size distribution using seven (four hydrophilic and three hydrophobic) log-normal modes. The aerosol number concentration and mass for each component are prognostically calculated with a constant geometric standard deviation of the aerosol size distribution. Uniform composition is maintained within modes (internal mixing), but compositional variations are allowed across different modes (external mixing). This size distribution is given by

$$n(\ln r)=\mathop{\sum }\limits_{i=1}^{7}\frac{{N}_{i}}{\sqrt{2\pi }\ln {\sigma }_{i}}\exp \left(-\frac{{(\ln r-\ln {\widetilde{r}}_{i})}^{2}}{2{\ln }^{2}{\sigma }_{i}}\right),$$

where each mode (*i*) is defined by the number concentration (*N*_*i*_), number median radius $({\widetilde{r}}_{i})$, and geometric standard deviation (*σ*_*i*_). The four hydrophilic modes encompass the entire aerosol size spectrum: (i) nucleation (<10 nm), (ii) Aitken (10–100 nm), (iii) accumulation (100–1000 nm), and (iv) coarse (>1000 nm). Similarly, the three hydrophobic modes span the same size range, corresponding to the Aitken, accumulation, and coarse modes^76^. In our simulations, *σ* = 1.59 for the nucleation mode, *σ* = 1.59 for the Aitken hydrophilic and hydrophobic modes, *σ* = 1.49 for the accumulation hydrophilic and hydrophobic modes, and *σ* = 1.7 for the coarse hydrophilic and hydrophobic modes^88^. To focus our analysis on specific size ranges, we integrate the log-normal distribution over the desired size intervals. This aerosol size distribution is evaluated in ref.^76^

Coagulation is described according to Vignati et al. ^89^, with coagulation coefficients calculated for Brownian motion based on the original work of Fuchs^90^. In GMXe, the coagulation matrix manages varying numbers of species per mode. Coagulation results in the transfer of aerosol particles from smaller to larger modes and from hydrophobic to hydrophilic modes. GMXe assumes that when two particles from the same mode coagulate, they form a particle within that mode, whereas coagulation of particles from different modes results in one in the larger mode. Coagulation between hydrophilic and hydrophobic modes produces a particle in the larger hydrophilic mode.

In ISORROPIA-II, the aerosol can inhabit either a thermodynamically stable state (precipitating salts when the aqueous solution phase attains saturation with respect to them) or a metastable state (aerosols predominantly composed of an aqueous phase that remains supersaturated in relation to dissolved salts). The model addresses both forward and reverse scenarios: either predicting gas/aerosol concentrations when the total (i.e. gas + aerosol) concentrations are known or deducing gas concentrations when aerosol concentration is given. In this study, we employ ISORROPIA-II in its metastable, forward mode^68^.

To address kinetic limitations in GMXe, gas/aerosol partitioning is calculated in two stages. First, the amount of gas-phase species that can kinetically condense onto the aerosol within a timestep is determined, assuming diffusion-limited condensation^89,90^. In the second stage, ISORROPIA-II redistributes the mass between the gas and aerosol phases. For low-volatility species, the total condensed amount matches the kinetic limit, while for semi-volatile species, only a fraction of the gas kinetically able to condense will partition into the aerosol phase based on thermodynamic considerations^68^.

## Supplementary information

Supplementary Information

## Data Availability

A permanent identifier (10.5281/zenodo.12743399) has been assigned in Zenodo under the ‘CERN CLOUD experiment community’, which includes the EMAC configuration files, namelist set-up, chemical mechanisms, and details on the emissions set-up. The full dataset shown in the figures is also available to ensure long-term availability and facilitate reproducibility.

## Code availability

The EMAC model is continuously developed and applied by a consortium of institutions. All affiliates of institutions that are part of the MESSy consortium are granted a license to use MESSy and access its source code. By signing the MESSy Memorandum of Understanding, institutions have the opportunity to become part of the MESSy consortium. Additional details are available on the MESSy consortium website (https://www.messy-interface.org). The results presented in this paper were produced with MESSy version 2.55.2 (DOI: 10.5281/zenodo.8360276). Details such as compiler settings are also included to achieve the highest possible degree of reproducibility.
