# Supplementary material for: The impact of ammonia on particle formation in the Asian Tropopause Aerosol Layer
Source: NPJ Clim Atmos Sci. 2024 Sep 12;7(1):215. doi: 10.1038/s41612-024-00758-3 (PMC11392815; doi:10.1038/s41612-024-00758-3)
Supplement: Supplementary file 1 — Supplementary Information [file 41612_2024_758_MOESM1_ESM.pdf]

Supplementary Information

Additional Model Evaluation

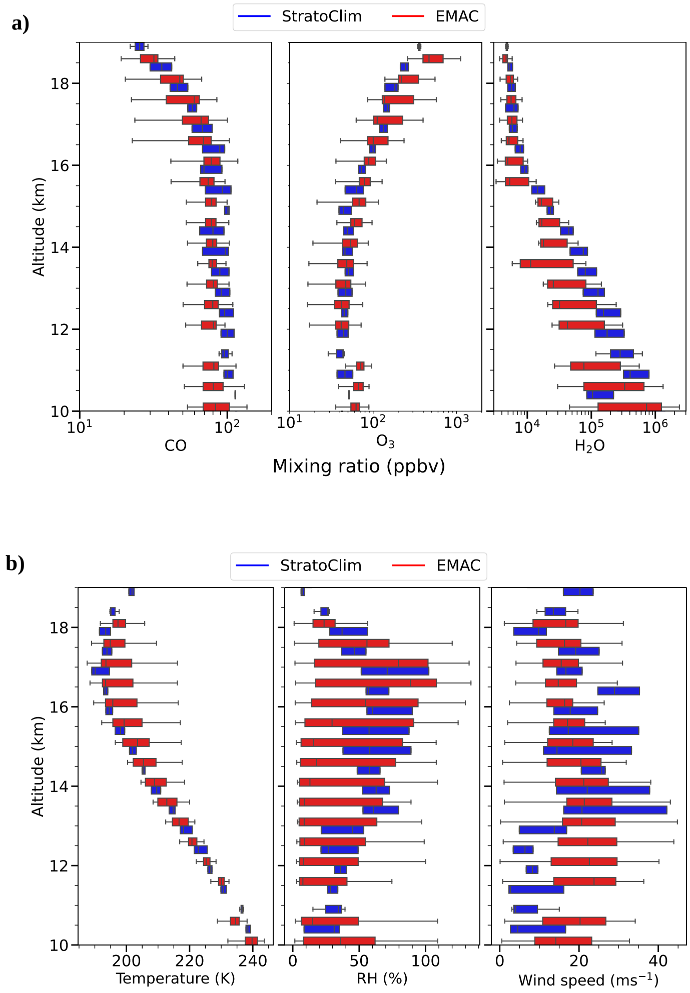

Supplementary Fig. 1 Further Evaluation of EMAC Model against StratoClim 2017 Observations. a) Vertical profiles of carbon monoxide (CO), ozone (O_3), and water vapour (H_2O) mixing ratios. **b)** Vertical profiles of atmospheric temperature, relative humidity (RH), and wind speed. Model data are compared to all eight flights during StratoClim between 27 July and 10 August 2017. Boxplots represent the distribution of observed (StratoClim) and modelled (EMAC) values. The central line of each boxplot denotes the median value, while the box boundaries indicate the interquartile range. The whiskers represent the range limits.

Anticyclone Evolution and Particle Number Concentration

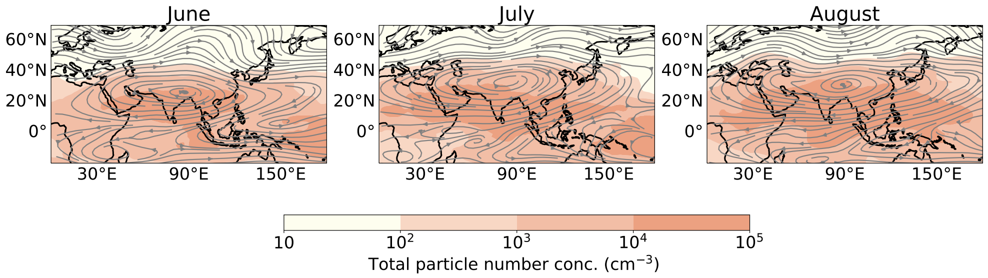

Supplementary Fig. 2 Anticyclone Evolution and Particle Number Concentration. The maps illustrate the monthly progression of the South Asian monsoon anticyclone and the associated distribution of particle number concentration as simulated by the EMAC model at 16 km altitude in the summer of 2017. The colour scale represents the total particle number concentration. All concentrations are calculated at ambient temperature and pressure. Grey arrows denote wind direction, highlighting how aerosols are confined near the anticyclone centre during the monsoon season.

Interannual Variability

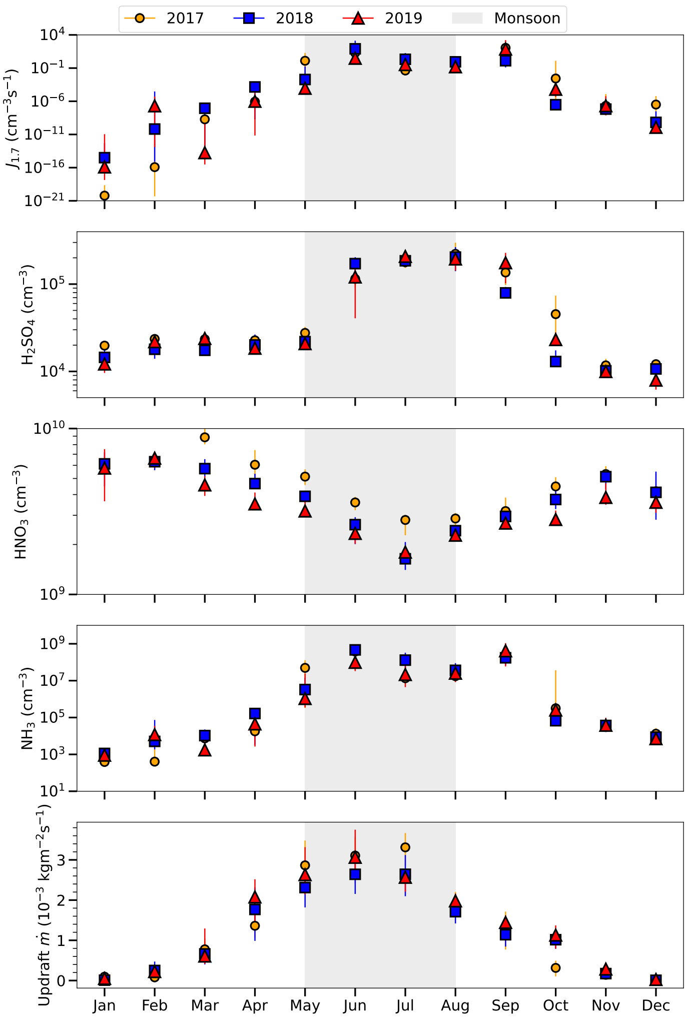

Supplementary Fig. 3 Temporal Variability. EMAC model simulation monthly median values for the nucleation rate at 1.7 nm ($J_{1.7}$), concentrations of precursor gases (H_2SO_4, HNO_3, NH_3), and updraft mass flux rate ($\dot{m}$) associated with deep convection across the South Asian monsoon region for 2017 (orange circles), 2018 (blue squares), and 2019 (red triangles). These simulations show the $J_{1.7}$ and gas concentrations at 16 km altitude, where the peak $J_{1.7}$ is reached within the ATAL. All concentrations are calculated at ambient temperature and pressure. Convective updrafts are shown at 5 km altitude, where vertical velocities are close to maximum. The vertical lines indicate the interquartile range. The grey region indicates the South Asian monsoon season.

Simulated NH_3 Surface Emissions

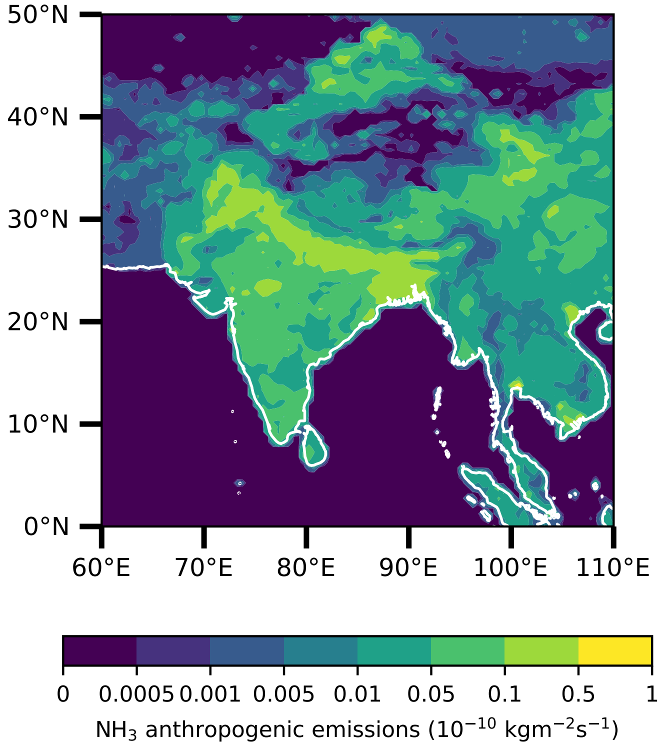

Supplementary Fig. 4 Simulated NH_3 Surface Anthropogenic Emissions in Summer 2017. This map depicts the simulated surface anthropogenic emissions of NH_3 across the South Asian monsoon region for the summer of 2017. Emissions data are derived using the Community Emissions Data System (CEDS) integrated into the EMAC model.
